# Species Redundancy and Niche Overlap: Mechanisms Maintaining Fish Community Function in Yangtze River Lakes in the Face of Lateral Hydrologic Connectivity Obstruction

**DOI:** 10.1002/ece3.72568

**Published:** 2025-12-11

**Authors:** Yuping Xu, Mengxuan Li, Dongmei Yu, Quehui Tang, Yiming Hu, Zhixin Zhou, Jianchao Liang, Huijian Hu

**Affiliations:** ^1^ Guangdong Key Laboratory of Animal Conservation and Resource Utilization, Institute of Zoology Guangdong Academy of Sciences Guangzhou China; ^2^ Guangzhou Aquatic Ecology and Construction Institution Guangzhou China; ^3^ South China Sea Fisheries Research Institute Chinese Academy of Fisheries Sciences Guangzhou China

**Keywords:** community stability, co‐occurrence network, river–lake disconnection, species redundancy hypothesis, Yangtze river

## Abstract

The obstruction of lateral hydrologic connectivity poses a significant threat to floodplain ecosystems, with fishes being particularly susceptible to the impacts of such disturbances. Existing research about the effects of human activities on fish communities in the Yangtze River basin primarily focused on measures of diversity such as species diversity, functional diversity, and beta diversity. This study goes beyond by examining patterns of fish species and functional diversity variation, as well as revealing the pattern of species disappearance and its implications for maintaining fish community structure and function. Our findings highlight the importance of understanding the role of fish species in maintaining ecosystem function. We revealed a significant decrease in both fish species richness and functional richness across temporal (1960s, 1980s, and 2000s) and spatial (connected lakes, partially connected lakes, and disconnected lakes) scales. Among the disappearing fish species, those belonging to small‐sized and large‐sized genera exhibited a higher frequency of disappearance compared to those in moderate‐sized genera. Nevertheless, the complexity of fish communities' co‐occurrence networks does not exhibit a significant decrease with a decrease in species/functional richness, potentially due to the presence of niche overlap species (e.g., coexistence species within a single genus in our study). Our findings support the species redundancy hypothesis in elucidating the mechanisms that uphold fish community function. This study underscores the importance of considering fish species correlation within a community for the effective management of ecosystems, biodiversity conservation, and ecological restoration.

## Graphical abstract

We revealed a significant decrease in both fish species richness and functional richness across temporal (1950s, 1980s, and 2000s) and spatial (connected lakes, partially connected lakes, and disconnected lakes) scales. Nevertheless, the complexity of fish communities' co‐occurrence networks does not exhibit a significant decrease with a decrease in species/functional richness, potentially due to the presence of niche overlap species.

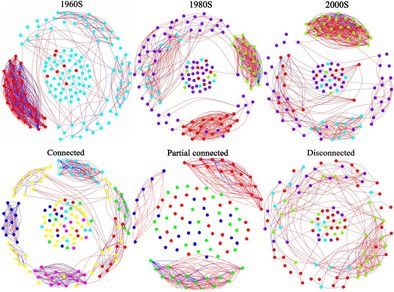

## 1 Introduction

The heightened interest in the impact of biodiversity on ecosystem stability is driven by the rapid acceleration of species extinctions, as evidenced by numerous studies (Ehrlich and Ehrlich 1981; Ehrlich and Wilson 1991; Wilson 1989; Wilson and Peter 1988). Ecosystem stability is contingent upon its ability to resist interference and capacity for recovery. It has been posited that greater stability of aggregate community properties with increasing diversity stems from the heightened likelihood of species or functional groups being present to adequately compensate for those affected by disturbances (Naeem and Li 1997; Tilman et al. 1996).

The stability of an ecosystem is contingent upon both the diversity of its community members (i.e., species diversity) and the various associations that may arise among coexisting members within the community (MacArthur 1955; Neutel et al. 2002). These associations can vary in nature, encompassing a spectrum from negative to positive, weak to strong, and nonsignificant to significant. Numerous theoretical frameworks have been proposed to explain the maintenance of species diversity and ecosystem stability, such as the diversity–stability hypothesis (MacArthur 1955), rivet‐popping hypothesis (Ehrlich and Ehrlich 1981), species redundancy hypothesis (Walker 1992), idiosyncratic hypothesis (Lawton 1994), and keystone species hypothesis (Sala et al. 1996). The redundancy hypothesis, as posited by Walker (1992), has emerged as a significant concept in elucidating the ecological significance of biodiversity. This hypothesis suggests that within a functional group comprising numerous species, functional redundancy is often observed. Specifically, certain species within the group exhibit similar or identical traits and demonstrate asynchronous reactions to environmental fluctuations, or exhibit temporal niche differentiation (Walker 1992; Elmqvist et al. 2003), thereby contributing to the preservation of system stability in the face of disturbances (Naeem 1998; Thibaut et al. 2012).

Theoretical research suggests that ecological networks characterized by weak correlations exhibit greater stability compared to networks with strong correlations (Coyte et al. 2015; McCann and Hastings 1998; Neutel et al. 2002). Additionally, compartmentalization and the prevalence of negative correlations have been found to enhance network stability in the face of disturbances (Rooney et al. 2006; Stouffer and Bascompte 2011). Therefore, communities characterized by a high prevalence of positive connections among members are considered to exhibit instability, as individuals within these communities may collectively react to changes in their environment, leading to positive feedback and co‐oscillation (Coyte et al. 2015). Conversely, negative connections may serve to stabilize co‐oscillation within communities and enhance the resilience of their networks (Coyte et al. 2015).

Floodplains are recognized as highly productive ecosystems within the global landscape, particularly in the context of inundation areas. The Yangtze River, ranked as the third longest river globally, exhibits lateral hydrologic connectivity (referring to the connection between the main channel and adjacent floodplain water bodies, like lakes, wetlands, and secondary channels) with numerous shallow lakes under natural conditions, forming what is known as “River‐Lake Connected Systems.” (Liu and Wang 2010; Xie 2007; Amoros and Bornette 2002). The periodic fluctuations in hydrology within this intricate system create a variety of habitats and abundant food sources for aquatic organisms, ultimately contributing to the high levels of species diversity found in this environment (Liu and Wang 2010; Revenga et al. 2005; Tockner and Stanford 2002; Ward et al. 1998). The river basin has a lengthy history of land use and has undergone substantial environmental modifications due to rapid economic growth. The resultant human disturbances have had a significant impact on freshwater biodiversity within this river region (Dudgeon 2019). The obstruction of lateral hydrologic connectivity, specifically the disconnection between rivers and floodplains, is a result of the spatial and temporal management of freshwater resources by humans through various methods, such as lake reclamation and hydropower project construction. This obstruction poses a significant threat to floodplain ecosystems (Revenga et al. 2005; Saunders et al. 2002), impeding the exchange of matter, energy, and organisms between rivers and floodplains, diminishing habitat size and diversity, limiting access for fish to utilize abundant floodplain resources during their early life stages, and ultimately eliminating essential ecological functions, eliminating the benefits of intermediate disturbance of river dynamics on floodplain biological communities (Amoros and Bornette 2002; Liu and Wang 2010).

In the preceding decades, notably during the period of the 1960s to the 1980s, a majority of lakes within the Yangtze River basin have been isolated from the natural flow patterns of the river as a result of polderization (Huang and Jiang 2005), the establishment and utilization of various dam structures and sluice gates (Jiang et al. 2020; Liu and Wang 2010). Only Dongting Lake and Poyang Lake maintain a natural hydrological connection within the basin. Furthermore, lake area dramatically decreased (Huang and Jiang 2005; Zeng 1990). During the 1980s to 2000s, lake reclamation had basically ended, most of the lakes that have self‐recovery ability progressed to the convalescent phase. Nearly 55% of the lake area of Poyang Lake shrank during the 1950s to 1980s, but the shrinkage of lake area slowed down greatly during the 1980s to 2000s (Huang and Jiang 2005). More than 330 large levees have been built in Dongting Lake, covering an area of more than 1200 km^2^ during the 1950s to 1970s, until the launching of the policy “returning farmland to lakes” (Gu 2007). The disruption caused by prolonged and intense human interference has led to a continual reorganization of fish populations. Consequently, the coexistence of species and the stability of the aquatic communities, including their ability to resist and recover from disturbances, have been altered.

Fishes are one of the most vulnerable groups among the affected organisms (Moi et al. 2021; Villéger et al. 2017; Welcomme et al. 2006). Currently, the majority of research on the impact of human disturbances on fish communities within the Yangtze river basin has primarily examined the influence on fish diversity, including species diversity (Fu et al. 2003; Gao et al. 2019; Jiang et al. 2021; Xiong et al. 2023), functional diversity (Gao et al. 2019; Jiang et al. 2021; Zhang 2022), species population (Cheng et al. 2015; Gao et al. 2019), and beta diversity (Dai et al. 2020; Jiang et al. 2021). The vast majority of studies have shown that hydrological disconnection (e.g., dam construction) has serious combined effects on fishes mainly due to impoundment, habitat fragmentation and blocking, flow regime modification, and hypolimnetic discharges. The loss of endemic species, the blocking of migration routes, the recruitment of fish species that produce drifting eggs, the postponing of the start for annual spawning, the reduction of fish spawning and growth opportunities, etc., were all found. Besides, functional and phylogenetic richness also exhibited a sharp decline.

However, there is a notable gap in the literature regarding the effects of human disturbances on fish species co‐occurrence (Wu et al. 2019) and community function. In recent years, ecologists have increasingly employed network theory to examine community assembly processes, assess the resistance of communities to perturbations (i.e., community stability), and evaluate the significance of species in ecosystem functioning (Berry and Widder 2014; Kay et al. 2018; Gotelli and McCabe 2002).

This study seeks to examine the impact of prolonged and intense anthropogenic disturbance on the fish species community. Fish data from lakes with different connected degrees with the Yangtze River mainstem, which are located along the middle and lower sections of the Yangtze River were analyzed for three distinct time periods: 1950–1970 (during the reclamation phase), 1970–1990 (following the completion of reclamation), 1990–2010 (during the recovery phase).

Species richness and functional richness were compared across various lakes and time periods to assess the effects of anthropogenic disturbance on these metrics. Furthermore, we conducted an analysis to determine the specific species or genera that became extinct during various time periods in order to elucidate the patterns of decline in fish species diversity. Subsequently, we constructed fish species co‐occurrence networks to examine community assembly processes, assess the resistance of communities to perturbations, allowing us to infer the impact of human activity on fish community stability. Through this study, we aimed to contribute to a better understanding of the fish community response to external interference, with potential implications for the management, conservation, and restoration of fish populations.

## 2 Materials and Methods

### 2.1 Study Area and Data Collection

The Yangtze River floodplain, once home to numerous shallow lakes teeming with aquatic biodiversity (Wang and Dou 1998), has undergone significant changes since the 1960s, with the majority of these lakes becoming disconnected from the main river due to the construction of embankments and sluice gates. Today, only two large lakes, Lakes Poyang, and Dongting, remain connected to the Yangtze mainstem. Moreover, the lakes were also affected by various escalating human activities, including lake shrinkage, degradation of water quality, conversion of farmland, overfishing, and the practice of pen culture. This environmental degradation resulted in a significant decrease in biodiversity among aquatic organisms in these lakes, particularly in fish populations (Jiang et al. 2021; Le et al. 2010; Xiong et al. 2023).

**FIGURE 1:**
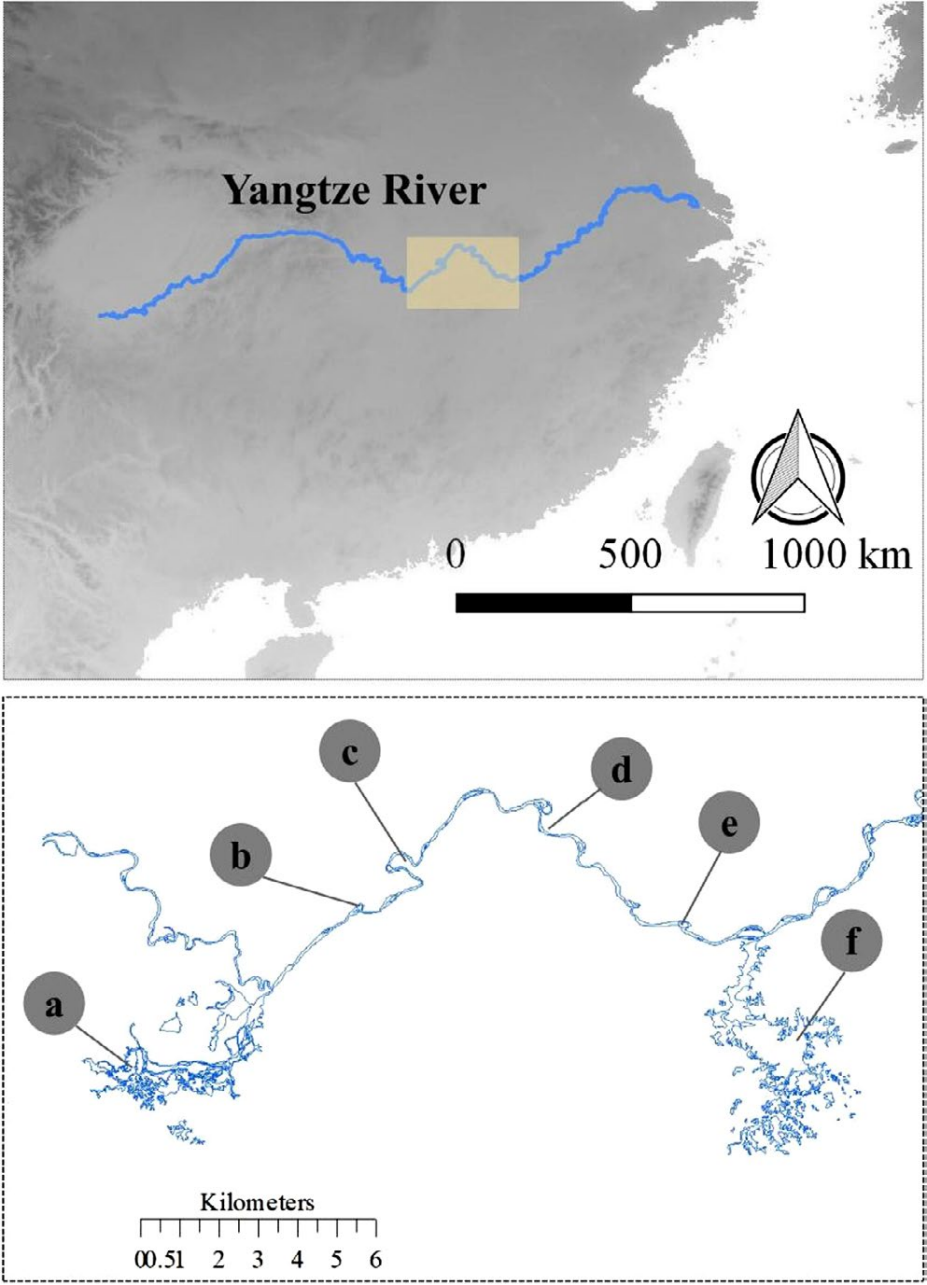
Six lakes' distribution in the Yangtze River basin, China. Connected lakes: a, West Dongting Lake, f, Poyang Lake; partially connected lake: b, Huanggai Lake; Disconnected Lakes: c, Xiliang Lake, d, Zhangdu Lake, e, Chi Lake.

In this study, our focus was on six lakes that have sufficient data on the temporal dynamics of fish fauna dating back to the 1960s, which was essential for subsequent analysis (Figure 1). In order to ensure the ecological representativeness of the floodplain system for the studied lakes, we followed a few selection principles: (1) connected lakes must be included, that is, Poyang Lake and Dongting Lake. (2) The disconnected lakes were connected with the Yangtze River in the past. (3) The distance between the lake and the Yangtze River should not be so far (the straight‐line distance should be less than 20 km). (4) The selected lakes need to be the main lake and sublakes of the main lake locally. (5) The lake area should not be too small (more than 30 km^2^). These lakes exhibit a diverse range of morphometric characteristics, with lake surface areas ranging from 100 to 5180 km^2^ in the historical period (the period when the studied lakes and the Yangtze River were still connected, i.e., 1930s) and 37 to 3150 km^2^ in the current period (refer to Table S2). Due to the variable disconnection time caused by hydraulic projects in the floodplain, which persisted until the 1960s, and the availability of data for the six lakes, the data were categorized into three distinct periods: the reclamation period (1960s, 1950–1970), reclamation completion period (1980s, 1970–1990), and recovery period (2000s, 1990–2010). Furthermore, the six lakes belong to three connection degrees with the Yangtze River, that is, connected lakes (West Dongting Lake and Poyang Lake), partially connected lakes (Huanggai Lake), and disconnected lakes (Zhangdu Lake, Chi Lake, and Xiliang Lake). Connected lakes mean the lake and the Yangtze River are connected all year round. Partially connected lakes mean the lake and the Yangtze River are connected for part of the year (e.g., half a year for Huanggai Lake). Disconnected lakes mean the lake is completely cut off from the Yangtze River. Fish occurrences in the six lakes were compiled from various sources including scientific reports, books, online data, county annals, aquaculture annals, and yearbooks dating back to the 1960s (Table S1). The taxonomy of the fish species was updated in accordance with FishBase (Froese and Pauly 2022). For each period, all available data were gathered to build up exhaustive species lists. We combined fish species lists from different years across the six lakes to maximize species representation for three periods, minimizing potential discrepancies from various sources. This resulted in fish species presence/absence matrices for each period.

### 2.2 Calculation of Multifaceted Alpha Diversities

Species richness was used to estimate taxonomic alpha diversity for fish in six lakes over three time periods. Functional alpha diversity was assessed using functional richness, which measures the extent of niche space occupied (Mason et al. 2005; Schleuter et al. 2010). Ten functional traits were selected to measure functional richness, including feeding habit, mouth position, trophic level, habitat, migration type, body shape, maximum total length, egg ecological type, resilience, and vulnerability, following Schleuter et al. (2010) and Mason et al. (2005) (Table S3). Functional richness increases with the number of species, as more species typically expand the functional space. However, two communities with the same species count can have different functional richness if their species' traits are more clustered in one community. Functional richness is not weighted by species abundance. Furthermore, we calculated genus size (number of species per genus) and then analyzed its frequency pattern, which could reflect the disappearance or maintenance of species that have niche overlap.

### 2.3 Statistical Analysis

We firstly compared the temporal differences of fish species and functional richness among the three periods with paired *t*‐tests using *pairwise.t.test* functions in R. Then we used the regression analysis to test the relationship between genera size and frequency for disappeared fish species. Linear and quadratic regressions were performed for each dataset and were considered significant when the coefficients of determination had a *p* value < 0.05. The 95% confidence intervals on modeled coefficients were used to examine and interpret relationships for each significant variable.

Networks of species co‐occurrences indicated the species participating in potential antagonistic or facilitative correlations, and the frequency of these co‐occurrences in which each species is involved. We examined co‐occurrence patterns with species presence–absence matrices to analyze community assembly processes and community stability over three periods. Robust Spearman correlations (| r | > 0.6 and *p* < 0.05) among fish species were used as proxies for interspecific co‐occurrence strengths. These were plotted as a weighted network, in which species represented by nodes were connected by links whose thicknesses represented Spearman correlations among species. Topological parameters—number of edges, number of vertices, connectance, average degree, average path length, and modularity—are considered important indicators of community complexity and stability and were used to describe ecological networks at the community level (Berry and Widder 2014; Morueta‐Holme et al. 2015; Rocchi et al. 2017; Wu et al. 2019). Connectance was calculated as a measure that accounts for network size, k/n^2^, where k is the total number of positive co‐occurrence links, and n is the number of network nodes (Kay et al. 2018). Average degree highlights node connectivity by measuring the average number of connections per node. A higher average degree means a denser network, while a lower one indicates a sparser network. Average path length measures the mean distance between pairs of nodes with shorter paths indicating more direct connections among species. Shorter path lengths enable faster transmission of information, energy, or resources, crucial for ecosystem functions like stability. Modularity measures network division, with increasing values indicating more fragmentation (Newman 2006). Species in the same module are more connected (more co‐occurred) inside than those outside. Lower modularity indicates a more interconnected network with less compartmentalized species correlations. The clustering coefficient represents the complexity of the network and strong correlations among organisms. Networks with high clustering coefficients are more cohesive than networks with lower coefficients (Jordán et al. 2008; Rocchi et al. 2017; Wu et al. 2019).

All above statistical analyses were conducted in the software R (version 3.6.3). We calculated functional alpha diversity indices in packages FD (Laliberté et al. 2014) and vegan (Oksanen et al. 2018). The *t*‐test was conducted by package vegan (Oksanen et al. 2018). The network visualization and parameter calculations were performed using the R package igraph (Csardi and Nepusz 2005).

## 3 Results

In the first period, before the loss of river–lake connectivity, 148 species belonging to 85 genera and 23 families were recorded across the 6 studied floodplain lakes. In the second period, there were 139 species, belonging to 83 genera and 23 families. In the third period, there were 123 species, belonging to 71 genera and 19 families. Overall, 25 species, 14 genera, and 4 families were extirpated from the species pool through time (Table S4).

### 3.1 Spatiotemporal Changes in Species and Functional Diversity of Fish Assemblages

**FIGURE 2:**
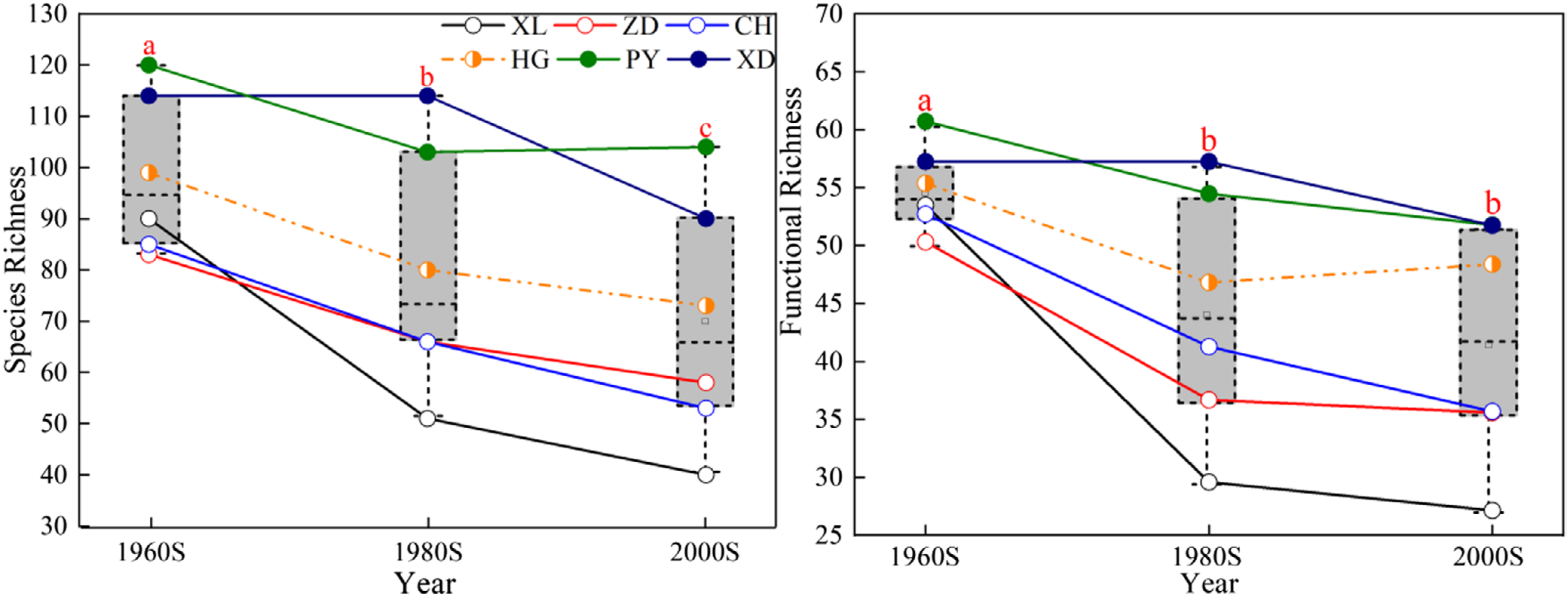
Temporal variations of species richness (left) and functional richness (right) for the six lakes across three periods. XD, West Dongting Lake; HG, Huanggai Lake; XL, Xiliang Lake; ZD, Zhangdu Lake; CH, Chihu Lake; PY, Poyang Lake. Different letters mean significant difference among gradients at the 0.05 level.

Both species richness and functional richness (Figure 2) decreased over time, with significant differences between the 1960s and 2000s (*p* = 0.002 for species richness, *p* = 0.009 for functional richness), and species richness in the 1980s was significantly higher than that in the 2000s (*p* = 0.028). But there was no significant difference in functional richness between the 1980s and 2000s (*p* = 0.064, Table S5). River‐disconnected lakes (XL, ZD, CH) experienced the largest declines in both species richness and functional richness, while river‐connected lakes (PY, XD) showed the smallest decreases. River‐partially connected lakes (HG) had intermediate declines.

### 3.2 Disappearance of Fish Species and Genera

During the study period, species loss mostly occurred, and rarely occurred in species gain. No species gain and only species loss occurred from the 1960s to the 1980s, and only three species gained from the 1980s to the 2000s, that is, *Ctenogobius cliffordpopei*, *Paracheilognathus imberbis* , *Rhodeus light*.

**FIGURE 3:**
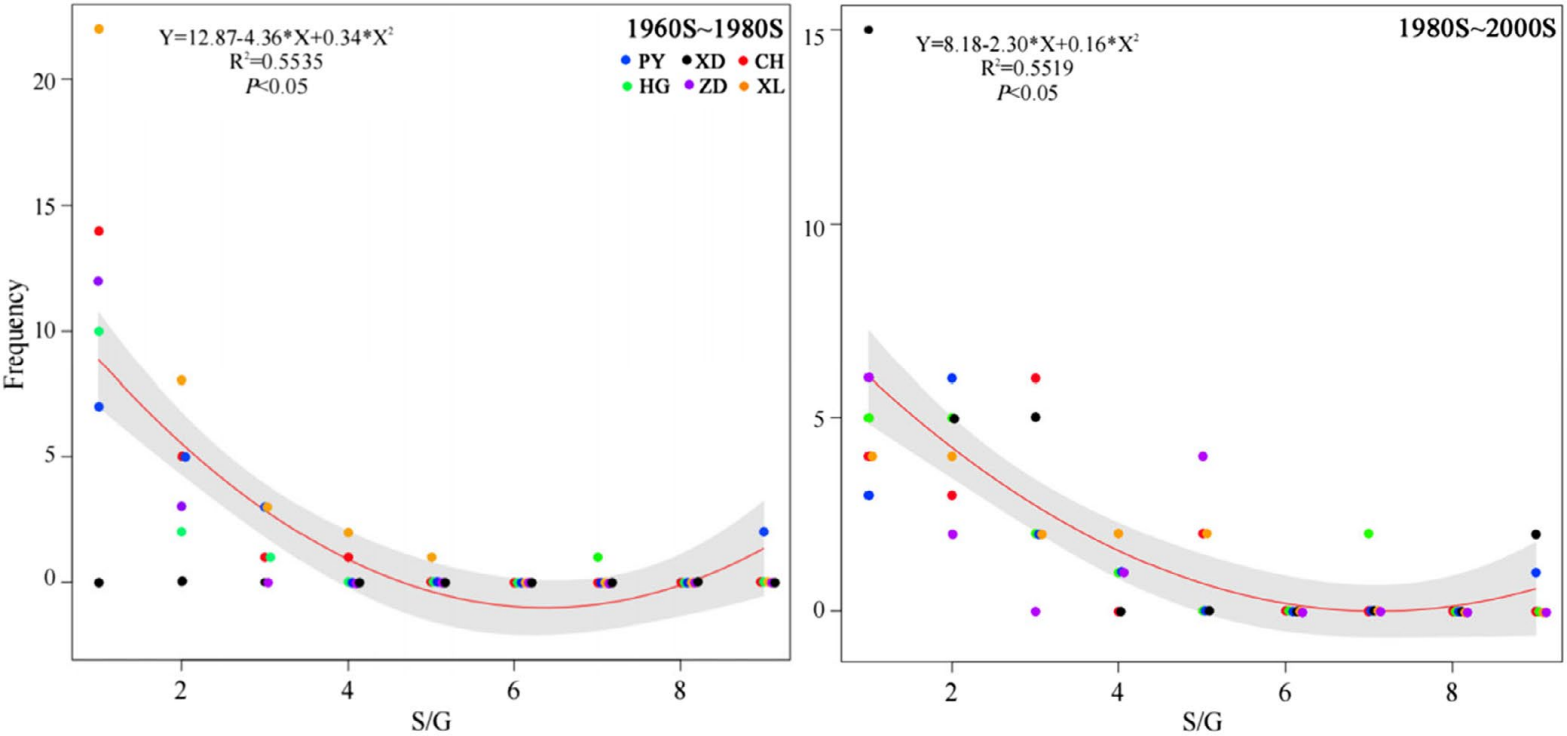
Size–frequency relationship of genera for disappeared species. S, Species richness; G, genera; S/G, species number per genera; referring to genera size. Frequency: The number of specific sized genera that disappeared. Different colors refer to different lakes.

Figure 3 illustrates the variability in genus size (number of species per genus) due to species loss. Frequency refers to the number of specific‐sized genera that disappeared in the 1980s and 2000s, calculated for each lake and period. A concave pattern was observed in the size–frequency relationship. Both large‐sized and small‐sized genera had higher frequencies than moderate‐sized genera in the 1980s and 2000s. It is undeniable that the disappearance of genera mainly occurred in small‐sized genera. Of those, six genera are migratory (i.e., *Ochetobius*, *Luciobrama*, *Anguilla*, *Macrura*, *Psephurus*, *Acipenser*), accounting for 43% of all migratory genera recorded in the six lakes.

Aside from a few vanished genera, the sizes of 30 genera listed in Table 1 and Table S6 have declined, particularly among larger genera like *Acheilognathus* and *Pelteobagrus*, which shrank across all six lakes and three periods (Table S6). Most size reductions were seen in small‐sized genera. The highest variation in genera occurred in Poyang Lake during the 1980s, followed by Xiliang Lake.

**TABLE 1:** Pattern of variability for genera size from the 1960s to the 1980s and from the 1980s to the 2000s.

Genera size	PY		CH		ZD		XL		HG		XD	
	1960S~1980S	1980S~2000S	1960S~1980S	1980S~2000S	1960S~1980S	1980S~2000S	1960S~1980S	1980S~2000S	1960S~1980S	1980S~2000S	1960S~1980S	1980S~2000S
**2**	12	6	5	6	4	5	8	0	1	2	0	4
**3**	2	1	3	4	1	0	3	2	2	2	0	4
**4**	2	1	0	0	0	1	2	2	0	1	0	0
**5**	0	1	0	1	0	1	1	1	0	0	0	0
**6**	0	0	0	0	0	0	0	0	0	0	0	0
**7**	0	0	0	0	0	0	0	0	1	1	0	0
**8**	1	1	0	0	0	0	0	0	0	0	0	1

*Note:* Disappeared species were not included. Genera size in this table means the starting genera size when species disappeared within the genera. Different colour shades indicate direct comparison between the different periods.

### 3.3 Co‐Occurrence Networks of Fish Communities

**FIGURE 4:**
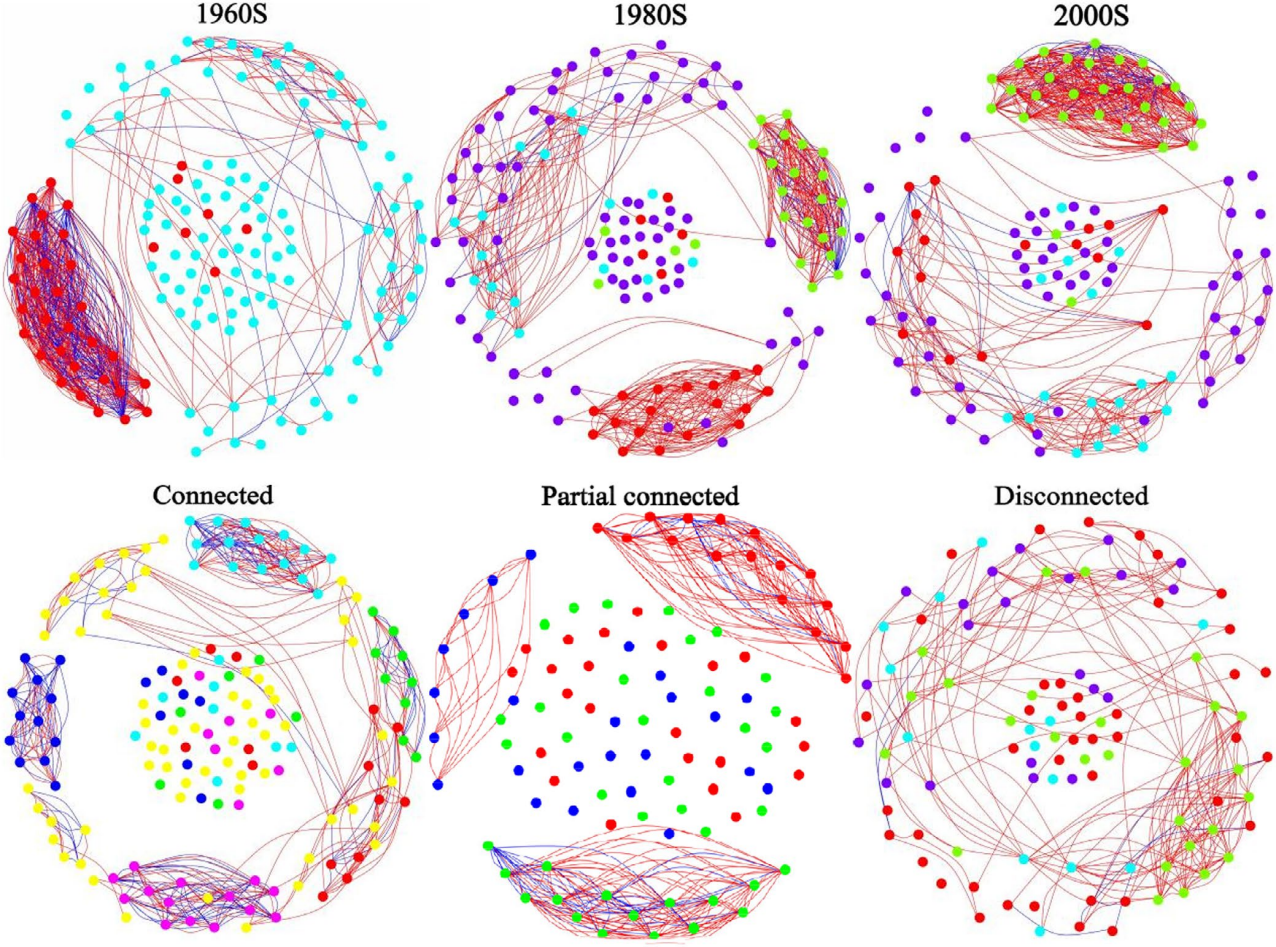
Fish co‐occurrence networks over time (**up**) and connection degree (**down**). Color of nodes indicates species from the same module in each network. Significant Spearman correlations (| r | > 0.6 and *p* < 0.05) are represented as edges, with red and blue edges denoting positive and negative correlations, respectively. The edge transparency corresponds to the strength of the correlation coefficients. Nodes of different colors represent distinct modules. **Connected**: Fish co‐occurrence networks of river‐connected lakes, including West Dongting Lake and Poyang Lake; **Partial connected**: Fish co‐occurrence networks of river‐partial connected lakes, that is, Huanggai Lke; **Disconnected**: Fish co‐occurrence networks of river‐disconnected lakes, including Lake Xiliang, Zhangdu and Chihu.

Networks for three periods and varying Yangtze River connections were constructed and analyzed for topological parameters (Figure 4 and Table 2). From the perspective of temporal variation, the fish network in the 1960s exhibited the highest clustering coefficient (0.77, Figure 4, a and Table 2), suggesting that fish species correlation was most robust during this period. The average path length values were also the lowest in the 1960s network (1.31, Figure 4, a and Table 2), indicating a compact network structure and strong interspecies co‐occurrence. Additional topological properties, such as lower connectance and modularity (Figure 4, a), and higher average degree, further imply that the fish network in the 1960s demonstrated a more interconnected structure. In the 1980s and 2000s, interspecies connections decreased, leading to longer path lengths and the formation of distinct modules, as indicated by higher connectance, average degree, and increased modularity (Figure 4, a).

**TABLE 2:** Topological properties of the fish community networks over time and connection degree.

Network indexes	1960S	1980S	2000S	Connected	Partial connected	Disconnected
Number of edges	581 (+385, −196)	494 (+447, −47)	546 (+484, −62)	401 (+242, −159)	261 (+218, −43)	212 (+204, −8)
Number of vertices	148	139	121	156	102	104
Connectance	0.018	0.023	0.037	0.010	0.021	0.020
Modularity	0.0616	0.0829	0.1074	0.0410	0.0287	0.0462
Average degree	100.73	73.88	64.30	102.90	91.59	53.71
Average path length	1.31	1.46	1.46	1.34	1.09	1.48
Clustering coefficient	0.77	0.64	0.66	0.74	0.93	0.62

Based on spatial variation analysis, the fish network in lakes partially connected to the Yangtze River exhibited the highest clustering coefficient (0.93, Table 2 and Figure 4, b) and the lowest average path length (1.09, Table 2 and Figure 4, b). This network showed the lowest modularity (0.0287, Table 2 and Figure 4, b), indicating higher interdependence among species. In contrast, fish networks in isolated lakes had fewer co‐occurrences and longer path lengths (1.48), with distinct, smaller modules (Figure 4, b), higher connectance (0.020), and average degree (53.71), but higher modularity (0.0462).

**FIGURE 5:**
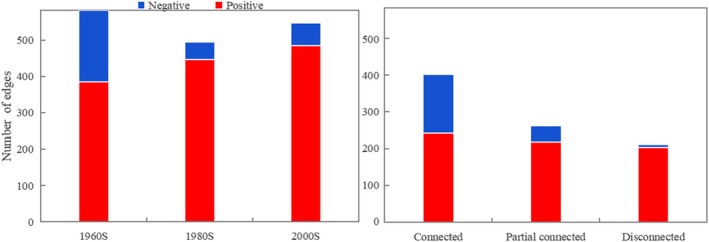
Number of positive (red bars) and negative correlation (blue bars) of the networks over time (left) and different degrees of connection with Yangtze River (right).

Fish networks across three time periods and three interconnected lakes linked to the Yangtze River underwent structural simplification. This was marked by a greater reduction in nodes and edges in networks isolated from the Yangtze River (see Figure 4, Figure 5, and Table 2). Additionally, most links in all three networks were positive (60.35% to 96.23%) (see Figure 5), indicating species tended to co‐occur rather than exclude each other. The rise in negative correlation from isolated to interconnected lakes supports the idea that reduced fish diversity promotes species co‐occurrence.

## 4 Discussion

### 4.1 The Decrease of Diversity and the Disappearance Pattern

The current study detected a clear decreasing trend of fish alpha diversity from the middle to lower reaches of the Yangtze River (see Figure 2). Results further illustrated how species diversity, functional diversity, genera size, and species correlation of fish assemblage respond to river–lake connectivity and human stress. In our study, species richness and functional richness exhibited the most significant decline in river‐disconnected lakes, while the least pronounced decline was observed in river‐connected lakes, specifically Lake West Dongting and Poyang. Previous research on other river floodplains has similarly reported that hydrological disconnection adversely impacts fish diversity (Burbano et al. 2020; Cooper et al. 2021; Couto et al. 2018; David et al. 2022; Santos et al. 2022). Firstly, limited or no river–floodplain connectivity often blocks pathways, hindering aquatic species' ability to disperse for spawning, foraging, and refuge, which leads to declines in biodiversity, particularly affecting migratory fishes like *Ochetobius*, *Luciobrama*, *Anguilla*, *Macrura*, *Psephurus*, and *Acipenser* in our study (Liermann et al. 2012; Paillex et al. 2009; Pettit et al. 2017). In addition, past land‐use practices have led to fewer wetlands and resultant habitat fragmentation, and exposure to high levels of agricultural and other stresses related to development (Xie and Chen 1999; Montgomery et al. 2020). Some studies found direct evidence that the process of river–lake fragmentation, which results in the decrease of water area and increase of lake isolation, has major impacts on the spatial structure of fish along the Yangtze River basin (Xie and Chen 1999; Wang, Hu, and Wang 2005; Wang, Lu, et al. 2005), like Zhangdu Lake (Wang, Hu, and Wang 2005), Huanggai Lake (Peng 2021). Overfishing, is another important threat that cannot be ignored in inland waters in the Yangtze River and elsewhere, as it often reduces fishery resources, biomass, abundance, and species richness (Chen et al. 2020; Liu et al. 2022). Li et al. (2013) concluded that overfishing, environmental pollution and hydropower project construction were the main reasons for the reduction of fish species richness in Dongting Lake, which were also the main causes of fishery resources decline in Poyang Lake (Wu et al. 2015).

Although genera are not inherently natural units, taxonomic classification systems partially reflect both the evolutionary process and the methodologies humans have employed to organize information (Holman 1985). As a result, higher taxonomic groups tend to encompass species that are, on average, significantly more phylogenetically related than would be anticipated by random distribution (Gaston 2000). From the disappearance pattern of fish genera, we found that large genera and small genera (e.g., monotypic genera) exhibit a higher frequency of species extinction. Closely related species are likely to exhibit high levels of similarity in ecological niches and spatial overlap (Zhao et al. 2019), leading to relatively strong interspecific competition for resources within genera (Armstrong and McGehee 1980; Chase and Leibold 2003; Elton 1946). Therefore, genera with a high species richness, situated at the extreme right of the abscissa axis, exhibit a higher frequency of species extinction compared to genera of moderate size when subjected to external perturbations and resource scarcity (Figure 3). This phenomenon may also contribute to the observed decline of certain large genera, such as *Acheilognathus* and *Pelteobagrus* (Table 2, Table S6). Moreover, the frequent extinction of species within small genera, positioned at the left extreme of the abscissa axis, can be attributed primarily to external perturbations and resource scarcity. This phenomenon is largely due to the widespread distribution of small genera within the community. Similarly, the overall decline in the size of small genera can be explained by the aforementioned factors. These elements likely influence the observed variation pattern in genera size.

### 4.2 Species Correlation Within Community

Community compositions are the outcome of species interaction, local environmental conditions, dispersal processes, and stochasticity (Vellend 2010). When facing external interference and resource limitations, interspecies connections within the community would change accordingly. Exploring species co‐occurrence can help determine disparate aspects of biotic communities and their variations (Araújo et al. 2011; Kay et al. 2018; Shukla and Bhat 2022). In 1980s and 2000s fish networks, the loss of species co‐occurrence, the decrease in link density and clustering as well as the increase in modularity, indicate a reduction in the resistance or function under external disturbance threats (Kay et al. 2018; Shukla and Bhat 2022). This is consistent with the decrease of SR and FR_ic_ exhibited in the 1980s and 2000s. Species extinction would be a direct result of environmental changes such as habitat loss, and the interdependence (link) between species would be lost when one of the partners disappears. However, the loss of the link itself due to rarity or phenological mismatch of the interacting partners (Berry and Widder 2014), may also potentially drive the extinction of one or both partners. Furthermore, the loss of species co‐occurrence could potentially also drive secondary species loss when one or more correlated partners depend strongly on the other (Berry and Widder 2014), thereby influencing the pattern and strength of interspecies direct/indirect correlation within the community. Community structure and function, diversity, and stability would accordingly be affected markedly (Dyer et al. 2010; Pocock et al. 2012).

It is worth mentioning that, although species richness and functional richness of partial‐connected lakes had a greater decrease than that in connected lakes (Figure 2), fish communities were much more aggregated in partial‐connected lakes (Table 2), presented as lower connectance and modularity. This suggested that the declines of species diversity or functional diversity do not necessarily cause the decrease of community complexity or stability. Previous studies (Kaiser‐Bunbury et al. 2010) found that the extinction of a species in co‐occurrence networks can lead to new links among surviving species. Additionally, the presence of niche‐overlapping species (e.g., fish within the same genera) ensures that interspecies correlations persist despite some extinctions, thereby maintaining network stability (Staniczenko et al. 2010).

Reclamation of lakes and hydropower projects on the Yangtze River have disconnected the river from its floodplain, blocking the exchange of matter, energy, and organisms. This has reduced habitat area and diversity, limiting fish access to floodplain resources during early life stages (Amoros and Bornette 2002; Liu and Wang 2010). It is a key reason for the decline in fish species and functional diversity.

We discovered that some species extinctions align with the species redundancy hypothesis, which suggests that multiple species can fulfill the same ecological role (Walker 1992; Burke et al. 2011), thus maintaining stability during disturbances (Naeem 1998; Elmqvist et al. 2003). Our study shows that larger genera are more frequently disappearing compared to moderate‐sized genera (Figure 3), indicating that species within the same genera are more prone to extinction due to their similar characteristics and strong interspecies competition. The disappearance pattern of this species aligns with the species redundancy hypothesis, which plays an important role in maintaining community stability. Our study found that positive interspecies links (Figure 5) increased over time (1960s, 1980s, 2000s) and across different lake connectivity levels (connected, partially connected, disconnected), indicating a trend toward cooperation rather than exclusion among fish. All the above conclusions also verified the fitness of the species redundancy hypothesis on fish diversity and community stability maintenance.

## 5 Conclusion

Human activities like river–floodplain disconnection and overfishing have significantly reduced fish species and functional diversity in Yangtze River lakes. However, our study indicates that interspecific relationships within fish communities remain stable and complex despite this decline. Extinctions within a community often follow a pattern where the loss of certain species is offset by others in the same genus performing similar roles, thereby maintaining the fish community's function. Our findings back the species redundancy hypothesis and have significant implications for understanding and predicting the ecological impacts of diversity loss and functional extinctions. Furthermore, our research underscores the significance of taking into account co‐occurrence among fish species in a given ecosystem for the efficient management of ecosystems, conservation of biodiversity, and restoration of ecological balance. Our findings help biologists use affordable diversity survey data to save time and resources, focusing on key species relationships for deeper exploration. This can enhance understanding of fish assemblage structures.

Restricted in the availability of long time series data, this study faces limitations due to the small sample size, hindering further exploration of fish co‐occurrence networks and environmental constraints. Future work should focus on increasing the sample size, particularly for the partially connected lakes, and utilizing more suitable tools like “cooccur” or “ecospat” to assess random effects and environmental factors in network patterns. Moreover, we will confirm our conclusion with a larger sample size of terrestrial animals, like waterbirds in the Yangtze River basin in the future.

## Author Contributions

**Yuping Xu:** conceptualization (lead), data curation (lead), formal analysis (lead), visualization (lead), writing – original draft (lead). **Mengxuan Li:** formal analysis (equal), funding acquisition (equal), project administration (equal). **Dongmei Yu:** data curation (equal), formal analysis (equal). **Quehui Tang:** formal analysis (equal), writing – review and editing (supporting). **Yiming Hu:** project administration (equal), supervision (equal). **Zhixin Zhou:** project administration (equal), supervision (equal). **Jianchao Liang:** data curation (equal), funding acquisition (equal), project administration (equal), supervision (lead), writing – review and editing (equal). **Huijian Hu:** data curation (equal), formal analysis (equal), project administration (equal), supervision (equal).

## Funding

This work was supported by the Guangzhou Water Science and Technology Project (GZSWKJ2022‐008), special funds for comprehensive Industrial Technology Innovation Center action of Guangdong Academy of Sciences (2022GDASZH‐2022010101), Guangdong Basic and Applied Basic Research Foundation (2021A1515110744), Forestry Technology Innovation Project of Guangdong Province (NO. 2023KJCX028), and Science and Technology Planning Project of Guangdong Province, China (2024B1212040009).

## Conflicts of Interest

The authors declare no conflicts of interest.

## Supporting information


**Table S1:** Literature that used to buildup fish species lists in three periods for the 6 studied lakes of Yangtze River floodplain.
**Table S2:** Information of the six lakes.
**Table S3:** Traits used to measure fish functional diversity.
**Table S4:** List of families (F), number of genera (G), and species (S) in the 6 studied lakes during the three periods.
**Table S5:** Results of paired *t‐*test for species richness (SR) and functional richness (FR) along time.
**Table S6:** Pattern of variability for genera size both from 1960s to 1980s and from 1980s to 2000s (Disappeared species were not included. 8–6 means that two species disappeared in the genera, and eight species per genera changed to six species per genera. “‐”indicates no change on the genera size. The same applies for other data of this table).
**Table S7:** Species lists of six studied lakes.

## Data Availability

The raw data for this work are available at the Supporting Information (Table S7).
