# Supplementary material for: Species Redundancy and Niche Overlap: Mechanisms Maintaining Fish Community Function in Yangtze River Lakes in the Face of Lateral Hydrologic Connectivity Obstruction
Source: Ecol Evol. 2025 Dec 11;15(12):e72568. doi: 10.1002/ece3.72568 (PMC12698507; doi:10.1002/ece3.72568)
Supplement: Supplementary file 1 — Table S1: Literature that used to buildup fish species lists in three periods for the 6 studied lakes of Yangtze River floodplain. Table S2: Information of the six lakes. Table S3: Traits used to measure fish functional diversity. Table S4: List of families (F), number of genera (G), and species (S) in the 6 studied lakes during the three periods. Table S5: Results of paired t‐test for species richness (SR) and functional richness (FR) along time. Table S6: Pattern of variability for genera size both from 1960s to 1980s and from 1980s to 2000s (Disappeared species were not included. 8–6 means that two species disappeared in the genera, and eight species per genera changed to six species per genera. “‐”indicates no change on the genera size. The same applies for other data of this table). Table S7: Species lists of six studied lakes. [file ECE3-15-e72568-s001.docx]

**Table S1 the Literature that used to buildup fish species lists in three periods for the 6 studied lakes of Yangtze** **River floodplain.**

| **No.** | **Category** | **Litterateurs** |
| --- | --- | --- |
| 1 | Journal publication | Li, J.Q., Wang, D.L., Ding, D.M., 2013. Research progress of fish resources in Dongting Lake. Journal of Anhui Agricultural Sciences, 41(9): 3898-3900. https://doi.org/10.13989/j.cnki.0517-6611. |
| 2 |  | Liu., F.R., 2008. Status of Fishery Resource and Countermeasure of Enhancement and Protection in Dongting Lake. Modern Fisheries Information. 17(8): 26-28. |
| 3 |  | Peng, P.B., Liu, S.L., Hu, H.J., Hu J.H., He M.Y., 2008. Monitoring the Dynamics of Fish Resources in Dongting Lake, Wetland Science & Management. 4(4): 17-20. https://doi.org/10.3969/j.issn.1673-3290.2008.04.007. |
| 4 |  | Hu, J.H., Hu, H.J., He, M.Y., Peng, P.B., Yang, D.D., 2006. Fish species diversity and its spatio-temporal variation in west Dongting Lake. Resources and Environment in the Yangtze Basin, 15(4): 434-441. |
| 5 |  | Liao, F.C., He, W., Huang, X.R., Jing, Q.L., He, X.C., 2002. Studies on present situation and change trend of Dongting Lake fishery resources and environment. Acta Hydrobiologica Sinica. 26(6): 623-627. |
| 6 |  | Liao, F.C., He, X.C., He, W., Wang, H.W., Xu D.P., 2006. Status and Protective Regulation Countermeasure in Fishery Resources and Its Environment of Dongting Lake. Journal of Yueyang Vocational and Technical College. 21(6):32-37. https://doi.org/10.3969/j.issn.1672-738X.2006.06.009. |
| 7 |  | Zhang, T.L., Li, Z.J., 2007. Fish resources and fishery utilization of Lake Poyang. Journal of Lake Sciences. 19(4): 434-444. https://doi.org/10.18307/2007.0412. |
| 8 |  | Ru, H.J., Liu, X.Q., Huang, X.R., Ning, Y. Z., Wang, H.Z., 2008. Diversity of fish species and its spatio-temporal variations in Lake Dongting, a large Yangtze-connected lake. Journal of Lake Sciences. 20(1): 93-99. https://doi.org/10.18307/2008.0114. |
| 9 |  | Tang, J.H., Qian, M.Q., 1979. Fish fauna in Dongting Lake. Freshwater Fisheries, Z1: 26-34. |
| 10 |  | Wang, L.M., Hu, H.J., Wang, D., 2005. Ecological impacts of disconnection from the Yangtze on fish resources in Zhangdu Lake. Resources and Environment in the Yangtze Basin. 14: 287-292. |
| 11 |  | Li, L.Y., Ni, Z.H., Li, Y.F., Wu, H.B., 2006. An investigation on the fishery resource and fish diversity of Zhangdu Lake. Freshwater Fisheries, 36(2): 18-23. |
| 12 | Dissertation | Hu, H.J., 2003. Ecological impacts on fish diversity after linking lakes disconnected from central Yangtze River. Beijing: Peking University. |
| 13 | Book | Zeng, X.Z., 1990. Fish Resources in the Yangtze River. Maritime Press, Beijing. |
| 14 |  | Dou, H.S., Jiang, J.H., 2000. Dongting Lake. University of Science and Technology of China Press, Beijing. |
| 15 |  | Local Chronicles Editorial Board of Puqi City, Hubei Province, 1995. Puqi County Annals . Haitian Press, Shenzhen. |
| 16 |  | Fish Research Centre, Institute of Hydrobiology of Hubei Province, 1976. Yangtze River Fish. Science Press, Beijing. |
| 17 |  | Local Chronicles Editorial Board of Jiayu County, Hubei Province, 1986. Xinzhou County Annals. Hubei Press, Wuhan. |
| 18 |  | Hunan Fisheries Science Institute, 1977. Hunan Fish, Hunan People Press, Hunan. |
| 19 |  | Yuan, Z.K., 2008. Resources and Environment of Dongting Lake Wetland. Hunan Normal University Press, Changsha, pp. 148–163. |
| 20 |  | Local Chronicles Editorial Board of Jiayu County, Hubei Province, 1993. Jiayu County Annals. Hubei Science and Technology Press, Wuhan. |
| 21 |  | Local Chronicles Editorial Board of Ruichang County, Jiangxi Province. 1990. Ruichang County Annals. Xinhua Press, Beijing. |
| 22 |  | Jin, B.X., Deng, Z.R., Li, X.M., 1992. Comprehensive Study of Jianghan Lakes Area. Hubei Science and Technology Publishing House, Wuhan. |
| 23 |  | Li, S.F., 2001. Research of biodiversity and conservation for important fish in Yangtze River.Shanghai Scientific and Technical Publishers, Shanghai. |
| 24 |  | Local Chronicles Editorial Board of Linxiang City, 1996. Linxiang City Annals. Hunan Publishing House, Changsha. |
| 25 |  | Meng, Q.W., 1996. Fish Taxonomy. China Agricultural Press, Beijing. |
| 26 |  | Changjiang Water Resources Commission of the Ministry of Water Resources, 1999. Atlas of the Changjiang River Basin. Sinomap press, Beijing. |
| 27 |  | Changjiang Water Resources Commission of the Ministry of Water Resources, 2001. Flood Control Atlas of the Changjiang River. Science Press, Beijing. |
| 28 |  | Fishes Editorial Board of Puqi, 1985. Fishes of Puqi County. |
| 29 |  | Bureau of Fisheries. Fishes of Xianning. |
| 30 | Website | Statistic Bureau of ChiBi, 2000. ChiBi Statistic yearbook. http://www.tjnjw.com. |
| 31 |  | Statistic Bureau of Jiayu, 2000. ChiBi Statistic yearbook. http://www.tjnjw.com. |
| 32 | Dataset | [dataset] Forestry Bureau of ChiBi City, 1998. Investigation Report of Wildlife Resources and Plant Distribution in ChiBi City. |
| 33 |  | [dataset] Forestry Bureau of Jiayu County, 1999. Investigation Report of Wildlife Resources in Jiayu County. |
| 34 |  | [dataset] Forestry Bureau of Jiujiang, 1998. Investigation Report of Jiujiang Wetlands. |
| 35 |  | [dataset] Statistic Bureau of Linxiang City, 2001. Linxiang Statistic yearbook. |
| 36 |  | [dataset] Forestry Bureau of Ruichang, 1999. Investigation Report of Wildlife Resources in Ruichang City. |

**Table S2 Information of the six lakes.**

| **Items** | **West Dongting** | **Poyang** | **Huanggai** | **Xiliang** | **Chihu** | **Zhangdu** |
| --- | --- | --- | --- | --- | --- | --- |
| Geographical location | E 111°57′—112°13′  N 28°53′—29°06′ | E 115º49′—116º46′  N 28º24′—29º46′ | E 113º29′—113º36′  N 29º37′—29º46′ | E 114º00′—114º10′  N 29º51′—30º02′ | E 115º37′—114º10′  N 29º41′—29º50′ | E 114°33′—115°45′  N 30°36′—30°48′ |
| Connection status | Connected | Connected | Partial-connected | disconnected | disconnected | disconnected |
| Disconnection reason | — | — | Dam and gate building | Gate building | Dam building | Dike building |
| Disconnection time | — | — | 1959 | 1935 | 1963 | 1964 |
| Present surface area (km^2^) | 300 | 3150 | 78 | 81 | 53 | 37 |
| Surface area in 1930S (km^2^) | 400 | 5180 | 288 | 159 | 100 | 155 |

**Table S3 Traits used to measure fish functional diversity.**

| **Classification of function** | **Functional trait** | **Type of functional traits** | **Rang or category** |
| --- | --- | --- | --- |
| Food acquisition | Feeding habits | Herbivorous, Omnivory, Sarcophagy | Binary variable |
|  | Mouth position | Sarcophagy, Terminal, Inferior | Categorical variable |
|  | Trophic level | / | Continuous variable (2.0-4.5) |
| Locomotion | Habitat | Demersal, Pelagic | Binary variable |
|  | Migration type | Non-migratory, Semi-migratory, Migratory | Binary variable |
|  | Maximum body length (cm) | / | Continuous variable (4.4-400) |
|  | Body shape | Inferior, Compressed, Anguilliform, Slightness, Sub-cylinder | Binary variable |
| Reproduction | Ecological type of eggs | Pelagic eggs, Demersal eggs, Adhesive eggs | Binary variable |
| Population dynamics | Resilience | High resilience, Medium resilience, Low resilience | Binary variable |
|  | Vulnerability | / | Continuous variable (1-100) |

**Table S4** List of families (F), number of genera (G) and species (S) in the 6 studied lakes during the three periods.

| **Items** | **West Dongting** | | | **Poyang** | | | **Huanggai** | | | **Xiliang** | | | **Zhangdu** | | | **Chihu** | | | **Summary** | | |
| --- | --- | --- | --- | --- | --- | --- | --- | --- | --- | --- | --- | --- | --- | --- | --- | --- | --- | --- | --- | --- | --- |
|  | S | G | F | S | G | F | S | G | F | S | G | F | S | G | F | S | G | F | S | G | F |
| **1960S** | 114 | 75 | 22 | 120 | 74 | 22 | 99 | 69 | 20 | 90 | 65 | 20 | 83 | 63 | 20 | 85 | 60 | 20 | 148 | 85 | 23 |
| **1980S** | 114 | 73 | 22 | 103 | 68 | 20 | 80 | 51 | 15 | 51 | 39 | 12 | 66 | 47 | 15 | 66 | 46 | 15 | 139 | 83 | 23 |
| **2000S** | 90 | 59 | 15 | 104 | 65 | 19 | 73 | 53 | 17 | 40 | 34 | 11 | 58 | 45 | 15 | 53 | 42 | 15 | 123 | 71 | 19 |

**Table S5** The results of paired T test for species richness (SR) and functional richness (FR) along time

| Paired groups | T value | df | P-value |
| --- | --- | --- | --- |
| SR(1960S-1980S) | 3.66 | 5 | 0.015 |
| SR(1980S-2000S) | 3.07 | 5 | 0.028 |
| SR(1960S-2000S) | 6.11 | 5 | 0.002 |
| FR(1960S-1980S) | 3.24 | 5 | 0.023 |
| FR(1980S-2000S) | 2.37 | 5 | 0.064 |
| FR(1960S-2000S) | 4.15 | 5 | 0.009 |

**Table S6** The pattern of variability for genera size both from 1960S to 1980S and from 1980S to 2000S (Disappeared species were not included. 8-6 means that two species disappeared in the genera, and eight species per genera changed to six species per genera. “-”indicates no change on the genera size. The same applies for other data of this table).

| **Lakes**  **Genera** | **PY** | | **CH** | | **ZD** | | **XL** | | **HG** | | **XD** | |
| --- | --- | --- | --- | --- | --- | --- | --- | --- | --- | --- | --- | --- |
|  | **1980S** | **2000S** | **1980S** | **2000S** | **1980S** | **2000S** | **1980S** | **2000S** | **1980S** | **2000S** | **1980S** | **2000S** |
| ***Abbottina*** | - | - | 2-1 | 2-1 | - | - | 2-1 | - | - | 2-1 | - | 3-2 |
| ***Acheilognathus*** | 8-6 | 8-5 | - | 5-3 | - | 4-3 | 5-3 | 5-2 | 7-6 | 7-4 | - | 8-7 |
| ***Acrossocheilus*** | 2-1 | - | - | - | - | - | - | - | - | - | - | - |
| ***Channa*** | - | - | - | 2-1 | - | - | - | - | - | - | - | - |
| ***Cobitis*** | 2-1 | - | - | - | 2-1 | 2-1 | 2-1 | - | - | - | - | 3-1 |
| ***Coilia*** | - | - | - | 2-1 | 2-1 | 2-1 | - | - | - | - | - | - |
| ***Ctenogobius*** | - | - | - | - | 2-1 | 2-1 | - | - | - | - | - | - |
| ***Cynoglossus*** | 3-1 | - | - | - | - | - | - | - | - | - | - | - |
| ***Distoechodon*** | - | - | - | - | - | 2-1 | 2-1 | - | - | - | - | - |
| ***Erythroculter*** | - | - | - | - | - | 5-1 | 4-3 | 4-2 | - | - | - | - |
| ***Fugu*** | 2-1 | - | 2-1 | - | - | - | - | - | - | - | - | - |
| ***Gobiobotia*** | - | - | - | - | - | - | - | - | - | - | - | 2-1 |
| ***Hemibarbus*** | 2-1 | 2-1 | - | - | - | - | - | - | - | - | - | - |
| ***Leiocassis*** | 2-1 | 2-1 | - | - | - | - | - | - | - | 2-1 | - | 2-1 |
| ***Leptobotia*** | 2-1 | 2-1 | - | - | - | - | - | - | - | - | - | 3-1 |
| ***Liobagrus*** | 2-1 | 2-1 | - | - | - | - | - | - | - | - | - | - |
| ***Macropodus*** | 2-1 | - | - | - | - | - | - | - | - | - | - | - |
| ***Megalobrama*** | - | 2-1 | - | - | - | - | 2-1 | - | - | - | - | - |
| ***Neosalanx*** | 3-2 | 3-2 | 2-1 | 2-1 | - | - | 2-1 | - | - | - | - | - |
| ***Parabotia*** | - | - | 2-1 | 2-1 | - | - | - | - | - | - | - | 2-1 |
| ***Pelteobagrus*** | 4-2 | - | 3-2 | 3-2 | - | - | 4-3 | 4-2 | - | 4-3 | - | - |
| ***Pseudobagrus*** | - | 4-2 | - | - | - | - | - | - | 2-1 | - | - | - |
| ***Pseudolaubuca*** | - | - | 2-1 | 2-1 | - | - | 2-1 | - | - | - | - | - |
| ***Rhinogobio*** | 2-1 | 2-1 | - | - | - | - | - | - | - | - | - | 2-1 |
| ***Rhodeus*** | 2-1 | - | 3-2 | 3-1 | 2-1 | - | 2-1 | - | - | - | - | - |
| ***Sarcocheilichthys*** | 4-2 | - | - | 3-2 | - | - | 3-2 | - | 3-2 | - | - | - |
| ***Saurogobio*** | 2-1 | - | - | - | - | - |  | 3-1 | 3-2 | 3-1 | - | 3-2 |
| ***Siniperca*** | - | - | - | 3-2 | 3-1 | - | 3-2 | 3-1 | - | - | - | - |
| ***Squalidus*** | 2-1 | - | - | - | - | - | 2-1 | - | - | - | - | - |
| ***Xenocypris*** | - | - | 3-2 | - | - | 2-1 | 3-1 | - | - | 3-2 | - | - |

**Table S7 Species lists of six studied lakes**

| **Lakes** | **West Dongting** | | | **Huanggai** | | | **Xiliang** | | | **Zhangdu** | | | **Chihu** | | | **Poyang** | | |
| --- | --- | --- | --- | --- | --- | --- | --- | --- | --- | --- | --- | --- | --- | --- | --- | --- | --- | --- |
| **Species** | XD1960 | XD1980 | XD2000 | HG1960 | HG1980 | HG2000 | XL1960 | XL1980 | XL2000 | ZD1960 | ZD1980 | ZD2000 | CH1960 | CH1980 | CH2000 | PY1960 | PY1980 | PY2000 |
| ***Acipenser sinensis*** | 1 | 1 |  | 1 |  |  | 1 |  |  | 1 |  |  | 1 |  |  | 1 |  |  |
| ***Psephurus gladius*** | 1 | 1 |  | 1 |  |  | 1 |  |  | 1 |  |  | 1 |  |  | 1 |  |  |
| ***Coilia brachygnathus*** | 1 | 1 | 1 | 1 | 1 | 1 | 1 |  |  | 1 | 1 | 1 | 1 | 1 | 1 | 1 | 1 | 1 |
| ***Coilia ectenes*** |  |  |  |  |  |  |  |  |  | 1 |  |  | 1 | 1 |  | 1 | 1 | 1 |
| ***Macrura reevesii*** | 1 | 1 |  | 1 |  | 1 | 1 |  |  | 1 |  |  | 1 |  |  | 1 | 1 | 1 |
| ***Hemisalanx brachyrostralis*** | 1 | 1 | 1 | 1 | 1 | 1 | 1 |  |  | 1 | 1 | 1 | 1 | 1 | 1 | 1 | 1 | 1 |
| ***Protosalanx hyalocranius*** | 1 | 1 | 1 | 1 |  |  | 1 |  |  |  |  |  | 1 |  |  |  | 1 | 1 |
| ***Neosalanx tangkahkeii*** | 1 | 1 | 1 | 1 | 1 | 1 | 1 |  |  | 1 | 1 | 1 | 1 | 1 | 1 | 1 | 1 | 1 |
| ***Neosalanx oligodontis*** | 1 | 1 | 1 | 1 | 1 | 1 | 1 | 1 | 1 |  |  |  | 1 |  |  | 1 | 1 | 1 |
| ***Neosalanx jordani*** |  |  |  |  |  |  |  |  |  |  |  |  |  |  |  | 1 |  |  |
| ***Anguilla japonica*** | 1 | 1 | 1 | 1 |  | 1 | 1 |  |  | 1 |  |  | 1 |  |  | 1 | 1 | 1 |
| ***Spinibarbus caldwelli*** | 1 | 1 |  |  |  |  |  |  |  |  |  |  |  |  |  | 1 | 1 | 1 |
| ***Zacco platypus*** |  |  |  |  |  |  |  |  |  |  |  |  |  |  |  | 1 | 1 | 1 |
| ***Phoxinus oxycephalus*** |  |  |  |  |  |  |  |  |  |  |  |  |  |  |  | 1 | 1 |  |
| ***Sinilabeo decorus*** | 1 | 1 |  | 1 | 1 |  |  |  |  |  |  |  |  |  |  |  |  |  |
| ***Acrossocheilus labiatues*** | 1 | 1 |  |  |  |  |  |  |  |  |  |  |  |  |  |  |  |  |
| ***Acrossocheilus formosanus*** |  |  |  |  |  |  |  |  |  |  |  |  |  |  |  | 1 |  |  |
| ***Acrossocheilus fasciatus*** |  |  |  |  |  |  |  |  |  |  |  |  |  |  |  | 1 | 1 |  |
| ***Onychostomas rara*** |  |  |  |  |  |  |  |  |  |  |  |  |  |  |  |  | 1 |  |
| ***Cyprinus carpio*** | 1 | 1 | 1 | 1 | 1 | 1 | 1 | 1 | 1 | 1 | 1 | 1 | 1 | 1 | 1 | 1 | 1 | 1 |
| ***Carassius auratus*** | 1 | 1 | 1 | 1 | 1 | 1 | 1 | 1 | 1 | 1 | 1 | 1 | 1 | 1 | 1 | 1 | 1 | 1 |
| ***Abbottina rivularis*** | 1 | 1 | 1 | 1 | 1 | 1 | 1 | 1 | 1 | 1 | 1 | 1 | 1 | 1 | 1 | 1 | 1 | 1 |
| ***Abbottina tungtingensis*** | 1 | 1 | 1 | 1 | 1 |  | 1 |  |  |  |  |  |  |  |  |  |  |  |
| ***Abbottina fukiensis*** | 1 | 1 |  |  |  |  |  |  |  |  |  |  | 1 |  |  |  |  |  |
| ***Platysmacheilus nudiventris*** | 1 | 1 |  | 1 |  |  |  |  |  |  |  |  |  |  |  |  |  |  |
| ***Pseudorasbora parva*** | 1 | 1 | 1 | 1 | 1 | 1 | 1 | 1 | 1 | 1 | 1 | 1 | 1 | 1 | 1 | 1 | 1 | 1 |
| ***Pseudorasbora elongata*** |  |  |  |  |  |  |  |  |  |  |  |  |  |  |  |  | 1 |  |
| ***Sarcocheilichthys sinensis*** | 1 | 1 | 1 | 1 | 1 | 1 | 1 | 1 | 1 | 1 | 1 | 1 | 1 | 1 | 1 | 1 | 1 | 1 |
| ***Sarcocheilichthys nigripinnis*** | 1 | 1 | 1 | 1 | 1 | 1 | 1 | 1 | 1 | 1 | 1 | 1 | 1 | 1 | 1 | 1 | 1 | 1 |
| ***Sarcocheilichthys parvus*** |  |  |  |  |  |  |  |  |  |  |  |  |  |  |  | 1 |  | 1 |
| ***Sarcocheilichthys kiangsiensis*** | 1 | 1 | 1 | 1 |  | 1 | 1 |  |  |  |  |  | 1 | 1 |  | 1 |  | 1 |
| ***Pseudogobio vaillanti*** | 1 | 1 | 1 | 1 | 1 |  | 1 |  |  | 1 | 1 |  | 1 |  |  |  |  |  |
| ***Gnathopogon imberbis*** |  |  |  |  |  |  |  |  |  |  |  |  |  |  |  | 1 |  |  |
| ***Squalidus sihuensis*** | 1 | 1 | 1 | 1 | 1 |  | 1 | 1 |  |  |  |  |  | 1 |  |  |  |  |
| ***Squalidus nitens*** |  |  |  |  |  |  |  |  |  |  |  |  |  |  |  |  |  | 1 |
| ***Squalidus wolterdstoiffi*** |  |  |  |  |  |  |  |  |  |  |  |  |  |  |  | 1 |  | 1 |
| ***Squalidus argentatus*** | 1 | 1 | 1 | 1 | 1 |  | 1 |  |  | 1 |  | 1 |  |  |  | 1 | 1 | 1 |
| ***Paracanthobrama guichenoti*** | 1 | 1 | 1 | 1 | 1 | 1 | 1 |  |  | 1 | 1 | 1 | 1 | 1 | 1 | 1 |  | 1 |
| ***Rhinogobio cylindricus*** | 1 | 1 |  |  |  |  |  |  |  |  |  |  |  |  |  | 1 | 1 |  |
| ***Rhinogobio typus*** | 1 | 1 | 1 | 1 | 1 |  | 1 |  |  | 1 | 1 |  |  |  |  | 1 |  | 1 |
| ***Coreius heterodon*** | 1 | 1 | 1 | 1 |  | 1 | 1 |  |  | 1 |  |  | 1 |  |  | 1 | 1 | 1 |
| ***Coreius septentrionalis*** |  |  |  |  |  |  |  |  |  |  |  |  |  |  |  | 1 |  |  |
| ***Hemibarbus labeo*** | 1 | 1 | 1 | 1 | 1 | 1 |  |  |  |  |  |  | 1 |  |  | 1 | 1 |  |
| ***Hemibarbus maculatus*** | 1 | 1 | 1 | 1 | 1 | 1 | 1 | 1 | 1 | 1 | 1 | 1 |  | 1 | 1 | 1 | 1 | 1 |
| ***Microphysogobio tungtingensis*** |  |  |  |  |  |  |  |  |  |  |  |  |  |  |  |  |  | 1 |
| ***Saurogobio dabryi*** | 1 | 1 | 1 | 1 | 1 | 1 | 1 | 1 | 1 | 1 | 1 | 1 | 1 | 1 | 1 | 1 | 1 | 1 |
| ***Saurogobio dumerili*** | 1 | 1 |  | 1 |  |  |  |  |  |  |  |  |  |  |  | 1 |  | 1 |
| ***Saurogobio gymnocheilus*** | 1 | 1 | 1 | 1 | 1 |  | 1 | 1 |  | 1 | 1 | 1 |  |  | 1 |  |  | 1 |
| ***Gobiobotia ichangensis*** | 1 | 1 | 1 |  |  | 1 | 1 |  |  |  |  |  |  |  |  | 1 | 1 |  |
| ***Gobiobotia longibarba*** | 1 | 1 |  |  |  |  |  |  |  |  |  |  |  |  |  |  |  |  |
| ***Elopichthys bambusa*** | 1 | 1 | 1 | 1 | 1 | 1 | 1 | 1 | 1 | 1 | 1 | 1 | 1 | 1 | 1 | 1 | 1 | 1 |
| ***Opsariichthys bidens*** | 1 | 1 | 1 | 1 | 1 | 1 | 1 |  |  | 1 |  |  |  | 1 |  | 1 | 1 | 1 |
| ***Squaliobarbus curriculus*** | 1 | 1 | 1 | 1 | 1 | 1 | 1 | 1 | 1 | 1 | 1 | 1 | 1 | 1 | 1 | 1 | 1 | 1 |
| ***Luciobrama macrocephalus*** | 1 | 1 | 1 | 1 |  | 1 | 1 |  |  |  |  |  |  |  |  | 1 | 1 | 1 |
| ***Ochetobius elongatus*** | 1 | 1 | 1 | 1 |  | 1 | 1 |  |  | 1 |  |  |  |  |  | 1 | 1 | 1 |
| ***Mylopharyngodon piceus*** | 1 | 1 | 1 | 1 | 1 | 1 | 1 | 1 | 1 | 1 | 1 | 1 | 1 | 1 | 1 | 1 | 1 | 1 |
| ***Ctenopharyngodon idellus*** | 1 | 1 | 1 | 1 | 1 | 1 | 1 | 1 | 1 | 1 | 1 | 1 | 1 | 1 | 1 | 1 | 1 | 1 |
| ***Sinibrama wui*** | 1 | 1 |  |  |  |  |  |  |  |  |  |  |  |  |  |  |  |  |
| ***Toxabramis swinhonis*** | 1 | 1 | 1 | 1 | 1 | 1 | 1 | 1 | 1 | 1 | 1 | 1 | 1 | 1 | 1 | 1 | 1 | 1 |
| ***Parabramis pekinensis*** | 1 | 1 | 1 | 1 | 1 | 1 | 1 | 1 | 1 | 1 | 1 | 1 | 1 | 1 | 1 | 1 | 1 | 1 |
| ***Megalobrama amblycephala*** | 1 | 1 | 1 | 1 | 1 | 1 | 1 | 1 | 1 | 1 | 1 | 1 | 1 | 1 | 1 | 1 | 1 | 1 |
| ***Megalobrama terminalis*** | 1 | 1 | 1 | 1 | 1 | 1 | 1 |  |  | 1 | 1 | 1 | 1 | 1 | 1 | 1 | 1 |  |
| ***Megalobrama skolkovii*** |  |  |  |  |  |  |  |  |  |  |  |  |  |  |  |  |  | 1 |
| ***Hemiculter bleekeri*** | 1 | 1 | 1 | 1 | 1 | 1 | 1 | 1 | 1 | 1 | 1 | 1 | 1 | 1 | 1 | 1 | 1 | 1 |
| ***Hemiculter leucisculus*** | 1 | 1 | 1 | 1 | 1 | 1 | 1 | 1 | 1 | 1 | 1 | 1 | 1 | 1 | 1 | 1 | 1 | 1 |
| ***Culter erythropterus*** | 1 | 1 | 1 | 1 | 1 | 1 | 1 | 1 | 1 | 1 | 1 | 1 | 1 | 1 | 1 | 1 | 1 | 1 |
| ***Culter dabryi*** |  |  |  |  |  |  |  |  |  |  |  | 1 |  |  |  |  |  |  |
| ***Erythroculter oxycephaloides*** | 1 | 1 | 1 | 1 | 1 | 1 | 1 |  |  | 1 | 1 |  |  |  |  | 1 | 1 | 1 |
| ***Erythroculter ilishaeformis*** | 1 | 1 | 1 | 1 | 1 | 1 | 1 | 1 | 1 | 1 | 1 |  | 1 | 1 | 1 | 1 | 1 | 1 |
| ***Erythroculter mongolicus*** | 1 | 1 | 1 | 1 | 1 | 1 | 1 | 1 | 1 | 1 | 1 |  | 1 | 1 | 1 | 1 | 1 | 1 |
| ***Erythroculter oxycephalus*** |  |  |  |  |  |  |  |  |  | 1 | 1 |  |  |  |  | 1 | 1 | 1 |
| ***Erythroculter dabryi*** | 1 | 1 | 1 | 1 | 1 | 1 | 1 | 1 |  | 1 | 1 | 1 | 1 | 1 | 1 | 1 | 1 | 1 |
| ***Pseudolaubuca sinensis*** | 1 | 1 | 1 | 1 | 1 | 1 | 1 | 1 |  | 1 | 1 | 1 | 1 |  |  | 1 | 1 | 1 |
| ***Pseudolaubuca engraulis*** | 1 | 1 | 1 | 1 | 1 | 1 | 1 |  |  | 1 | 1 | 1 | 1 | 1 | 1 | 1 | 1 | 1 |
| ***Xenocypris davidi*** | 1 | 1 | 1 | 1 | 1 | 1 | 1 |  |  |  | 1 | 1 | 1 |  |  | 1 | 1 | 1 |
| ***Xenocypris argentea*** | 1 | 1 | 1 | 1 | 1 | 1 | 1 | 1 |  | 1 | 1 | 1 | 1 | 1 |  | 1 | 1 | 1 |
| ***Xenocypris microlepis*** | 1 | 1 | 1 | 1 | 1 |  | 1 |  |  | 1 | 1 |  | 1 | 1 |  | 1 | 1 | 1 |
| ***Distoechodon tumirostris*** | 1 | 1 | 1 | 1 | 1 | 1 | 1 |  |  | 1 | 1 |  | 1 | 1 |  |  |  |  |
| ***Distoechodon hupeinensis*** |  |  |  |  |  |  | 1 | 1 | 1 | 1 | 1 | 1 |  |  | 1 |  |  |  |
| ***Pseudobrama simoni*** | 1 | 1 | 1 | 1 | 1 | 1 | 1 |  |  |  |  | 1 |  |  |  | 1 | 1 | 1 |
| ***Acheilognathus gracilis*** | 1 | 1 | 1 | 1 | 1 |  |  |  |  |  |  |  |  |  |  | 1 | 1 | 1 |
| ***Acheilognathus barbatus*** | 1 | 1 |  | 1 |  |  |  |  |  |  |  |  | 1 | 1 |  |  |  | 1 |
| ***Acheilognathus macropterus*** | 1 | 1 | 1 | 1 | 1 | 1 | 1 | 1 | 1 | 1 | 1 | 1 | 1 | 1 | 1 | 1 | 1 | 1 |
| ***Acheilognathus tonkinensis*** | 1 | 1 | 1 | 1 | 1 |  | 1 | 1 |  |  |  |  | 1 | 1 |  | 1 | 1 | 1 |
| ***Acheilognathus taenianalis*** | 1 | 1 | 1 | 1 | 1 | 1 | 1 |  | 1 | 1 | 1 | 1 | 1 | 1 | 1 | 1 |  |  |
| ***Acheilognathus hypselonotus*** | 1 | 1 | 1 | 1 | 1 | 1 | 1 |  |  | 1 | 1 |  |  |  |  | 1 | 1 |  |
| ***Acheilognathus polylepis*** | 1 | 1 | 1 |  |  |  |  |  |  |  |  |  |  |  |  |  |  |  |
| ***Acheilognathus chankaensis*** | 1 | 1 | 1 | 1 | 1 | 1 | 1 | 1 |  | 1 | 1 | 1 | 1 | 1 | 1 | 1 | 1 | 1 |
| ***Acheilognathus tabira*** |  |  |  |  |  |  |  |  |  |  |  |  |  |  |  | 1 | 1 | 1 |
| ***Acheilognathus elongatus*** |  |  |  |  |  |  |  |  |  |  |  |  |  |  |  | 1 |  |  |
| ***Paracheilognathus imberbis*** |  |  | 1 |  |  |  |  |  |  | 1 | 1 |  |  |  |  |  | 1 | 1 |
| ***Paracheilognathus himategus*** |  |  |  |  |  |  |  |  |  |  |  |  |  |  |  | 1 |  |  |
| ***Rhodeus sinensis*** | 1 | 1 | 1 | 1 | 1 | 1 | 1 | 1 | 1 | 1 | 1 | 1 | 1 |  | 1 |  |  |  |
| ***Rhodeus ocellatus*** | 1 | 1 | 1 |  |  |  |  |  |  | 1 |  | 1 | 1 | 1 |  | 1 |  | 1 |
| ***Rhodeus fangi*** |  |  |  |  |  |  |  |  |  |  |  |  |  |  |  |  | 1 | 1 |
| ***Rhodeus light*** |  |  | 1 | 1 | 1 | 1 | 1 |  |  |  |  |  | 1 | 1 |  | 1 | 1 | 1 |
| ***Hypophthalmichthys molitrix*** | 1 | 1 | 1 | 1 | 1 | 1 | 1 | 1 | 1 | 1 | 1 | 1 | 1 | 1 | 1 | 1 | 1 | 1 |
| ***Aristichthys nobilis*** | 1 | 1 | 1 | 1 | 1 | 1 | 1 | 1 | 1 | 1 | 1 | 1 | 1 | 1 | 1 | 1 | 1 | 1 |
| ***Myxocyprinus asiaticus*** | 1 | 1 |  | 1 |  |  | 1 |  |  | 1 |  |  | 1 |  |  |  | 1 |  |
| ***Rhynchocypris lagowskii*** |  |  |  |  |  |  |  |  |  |  |  |  |  |  |  | 1 | 1 |  |
| ***Lepturichthys fimbriata*** | 1 | 1 |  |  |  |  |  |  |  |  |  |  |  |  |  |  | 1 |  |
| ***Leptobotia pratti*** | 1 | 1 |  |  |  |  |  |  |  |  |  |  |  |  |  |  |  |  |
| ***Leptobotia elongata*** | 1 | 1 |  |  |  |  |  |  |  |  |  |  |  |  |  | 1 |  |  |
| ***Leptobotia taeniops*** | 1 | 1 | 1 | 1 | 1 |  | 1 | 1 | 1 | 1 | 1 | 1 | 1 | 1 |  | 1 | 1 | 1 |
| ***Cobitis taenia*** | 1 | 1 |  |  |  |  |  |  |  |  |  |  |  |  |  |  | 1 |  |
| ***Cobitis macrostigma*** | 1 | 1 |  | 1 |  |  | 1 |  |  | 1 |  |  | 1 |  |  | 1 | 1 | 1 |
| ***Cobitis sinensis*** | 1 | 1 | 1 |  | 1 | 1 | 1 | 1 |  | 1 | 1 | 1 |  |  | 1 | 1 |  | 1 |
| ***Botia superciliaris*** | 1 | 1 | 1 | 1 | 1 |  | 1 |  |  | 1 | 1 |  | 1 | 1 | 1 |  |  |  |
| ***Parabotia fasciata*** | 1 | 1 |  | 1 | 1 | 1 |  |  |  |  |  |  | 1 |  |  | 1 | 1 | 1 |
| ***Parabotia banarescui*** | 1 | 1 | 1 | 1 | 1 | 1 | 1 | 1 |  | 1 | 1 | 1 | 1 | 1 | 1 |  | 1 | 1 |
| ***Misgurnus anguillicaudatus*** | 1 | 1 | 1 | 1 | 1 | 1 | 1 | 1 | 1 | 1 | 1 | 1 | 1 | 1 | 1 | 1 | 1 | 1 |
| ***Paramisgumus dabryanus*** |  |  |  | 1 |  |  |  |  |  |  |  |  |  |  |  | 1 |  |  |
| ***Leiocassis longirostris*** | 1 | 1 | 1 | 1 | 1 | 1 | 1 |  |  | 1 |  |  | 1 |  |  | 1 | 1 | 1 |
| ***Leiocassis crassilabris*** | 1 | 1 |  | 1 | 1 |  |  |  |  |  |  |  |  |  |  | 1 |  |  |
| ***Pseudobagrus albomarginatus*** | 1 | 1 | 1 | 1 | 1 |  | 1 | 1 |  | 1 | 1 |  | 1 | 1 |  | 1 | 1 |  |
| ***Pseudobagrus ussuriensis*** | 1 | 1 | 1 | 1 |  |  |  |  |  |  |  |  |  |  |  | 1 | 1 |  |
| ***Pseudobagrus tenuis*** |  |  |  |  |  |  |  |  |  |  |  |  |  |  |  | 1 | 1 | 1 |
| ***Pseudobagrus pratti*** |  |  |  |  |  |  |  |  |  |  |  |  |  |  |  | 1 | 1 | 1 |
| ***Pseudobagms emarginatus*** |  |  |  |  |  |  |  |  |  |  |  |  |  |  |  |  | 1 |  |
| ***Mystus macropterus*** | 1 | 1 |  |  |  |  |  |  |  | 1 |  |  | 1 |  |  | 1 | 1 | 1 |
| ***Pelteobagrus vachelli*** | 1 | 1 | 1 | 1 | 1 | 1 | 1 | 1 |  | 1 | 1 | 1 | 1 | 1 | 1 | 1 |  | 1 |
| ***Pelteobagrus nitidus*** | 1 | 1 | 1 | 1 | 1 |  | 1 | 1 | 1 |  |  |  | 1 | 1 | 1 | 1 |  | 1 |
| ***Pelteobagrus fulvidraco*** | 1 | 1 | 1 | 1 | 1 | 1 | 1 | 1 | 1 | 1 | 1 | 1 | 1 | 1 | 1 | 1 | 1 | 1 |
| ***Pelteobagrus eupogon*** | 1 | 1 | 1 | 1 | 1 | 1 | 1 |  |  |  |  |  | 1 |  |  | 1 | 1 | 1 |
| ***Silures asotus*** | 1 | 1 | 1 | 1 | 1 | 1 | 1 | 1 |  | 1 | 1 | 1 | 1 | 1 | 1 | 1 | 1 | 1 |
| ***Silurus soldatovi*** | 1 | 1 | 1 |  |  |  |  |  |  |  |  |  |  |  |  | 1 | 1 | 1 |
| ***Clarias fuscus*** |  |  |  |  |  |  |  |  |  |  |  |  |  |  |  | 1 | 1 | 1 |
| ***Liobagrus nigricauda*** |  |  |  |  |  |  |  |  |  |  |  |  |  |  |  | 1 |  | 1 |
| ***Liobagrus styani*** |  |  |  |  |  |  |  |  |  |  |  |  |  |  |  |  | 1 | 1 |
| ***Liobagrus marginatus*** |  |  |  |  |  |  |  |  |  |  |  |  |  |  |  |  | 1 |  |
| ***Liobagrus anguillicauda*** |  |  |  |  |  |  |  |  |  |  |  |  |  |  |  | 1 | 1 |  |
| ***Glyptothorax sinense*** | 1 | 1 |  | 1 |  |  |  |  |  |  |  |  |  |  |  | 1 | 1 | 1 |
| ***Oryzias latipes*** | 1 | 1 |  | 1 | 1 | 1 | 1 | 1 |  | 1 | 1 |  | 1 |  |  | 1 |  | 1 |
| ***Hemirhamphus kurumeus*** | 1 | 1 | 1 |  | 1 | 1 | 1 | 1 | 1 | 1 | 1 | 1 | 1 | 1 | 1 | 1 | 1 | 1 |
| ***Monopterus albus*** | 1 | 1 | 1 | 1 | 1 | 1 | 1 |  | 1 | 1 | 1 | 1 | 1 | 1 | 1 | 1 | 1 | 1 |
| ***Coreosiniperca roulei*** | 1 | 1 | 1 | 1 |  |  |  |  |  |  |  |  |  |  |  | 1 | 1 | 1 |
| ***Siniperca chuatsi*** | 1 | 1 | 1 | 1 | 1 | 1 | 1 |  | 1 | 1 |  | 1 | 1 | 1 | 1 | 1 | 1 | 1 |
| ***Siniperca kneri*** | 1 | 1 | 1 | 1 | 1 | 1 | 1 | 1 | 1 | 1 | 1 | 1 | 1 | 1 | 1 | 1 | 1 | 1 |
| ***Siniperca undulata*** |  |  |  |  |  |  |  |  |  |  |  |  |  |  |  |  | 1 | 1 |
| ***Siniperca scherzeri*** | 1 | 1 | 1 | 1 | 1 | 1 | 1 | 1 |  | 1 | 1 | 1 | 1 | 1 |  | 1 | 1 | 1 |
| ***Hypseleotris swinhonis*** | 1 | 1 | 1 | 1 | 1 | 1 | 1 | 1 | 1 | 1 | 1 | 1 | 1 | 1 | 1 | 1 | 1 | 1 |
| ***Eleotris fusca*** |  |  |  |  |  |  |  |  |  |  |  |  |  |  |  | 1 |  |  |
| ***Odontobutis obscura*** | 1 | 1 | 1 | 1 |  | 1 | 1 | 1 | 1 | 1 | 1 | 1 | 1 | 1 | 1 | 1 | 1 | 1 |
| ***Ctenogobius giurinus*** | 1 | 1 | 1 | 1 | 1 | 1 | 1 | 1 | 1 | 1 | 1 | 1 | 1 | 1 | 1 | 1 | 1 | 1 |
| ***Ctenogobius cliffordpopei*** |  |  | 1 |  |  |  |  |  |  | 1 |  |  |  |  |  |  | 1 | 1 |
| ***Rhinogobius similis*** | 1 | 1 |  | 1 | 1 | 1 | 1 |  |  |  |  |  | 1 | 1 |  |  |  |  |
| ***Mugilogobius myxodermus*** | 1 | 1 |  | 1 |  |  |  |  |  |  |  |  |  |  |  |  |  | 1 |
| ***Macropodus chinensis*** | 1 | 1 | 1 | 1 | 1 | 1 | 1 |  |  | 1 | 1 | 1 | 1 | 1 | 1 | 1 | 1 | 1 |
| ***Macropodus opercularis*** |  |  |  |  |  |  |  |  |  |  |  |  |  |  |  | 1 |  | 1 |
| ***Channa argus*** | 1 | 1 | 1 | 1 | 1 | 1 | 1 | 1 | 1 | 1 | 1 | 1 | 1 | 1 | 1 | 1 |  | 1 |
| ***Channa maculatus*** |  |  |  |  |  |  |  |  |  |  |  |  | 1 | 1 |  |  |  |  |
| ***Channa asiatica*** |  |  |  |  |  |  |  |  |  |  |  |  |  |  |  | 1 |  | 1 |
| ***Mastacembelus aculeatus*** | 1 | 1 | 1 | 1 | 1 | 1 | 1 | 1 | 1 | 1 | 1 | 1 | 1 | 1 | 1 |  | 1 | 1 |
| ***Mastacembelus armatus*** |  |  |  |  |  |  |  |  |  |  |  |  |  |  |  | 1 |  |  |
| ***Cynoglossus trigrammus*** |  |  |  |  |  |  |  |  |  | 1 |  |  | 1 |  |  | 1 |  |  |
| ***Cynoglossus gracilis*** |  |  |  |  |  |  |  |  |  |  |  |  |  |  |  | 1 | 1 |  |
| ***Cynoglossus semilaevis*** |  |  |  |  |  |  |  |  |  |  |  |  |  |  |  | 1 |  |  |
| ***Fugu obscurus*** | 1 | 1 |  | 1 |  |  | 1 |  |  | 1 |  |  | 1 | 1 |  | 1 | 1 |  |
| ***Fugu ocellatus*** |  |  |  |  |  |  |  |  |  |  |  |  | 1 |  |  | 1 |  |  |
